# Seroprevalence of Leptospirosis Among Patients With Acute Febrile Illness in a Tertiary Care Hospital in Northern Assam

**DOI:** 10.7759/cureus.105382

**Published:** 2026-03-17

**Authors:** Pinky Lahon, Mayuri Gogoi, Samrat Biswas

**Affiliations:** 1 Medical Microbiology, Tezpur Medical College and Hospital, Tezpur, IND; 2 Department of Microbiology, Jorhat Medical College and Hospital, Jorhat, IND

**Keywords:** acute febrile illness, enzyme-linked immunosorbent assay, igm antibody, leptospirosis, zoonotic disease

## Abstract

Introduction

Leptospirosis, a zoonotic disease with worldwide distribution, has emerged as an important public health concern. Rodents are the most common reservoir of infection. Humans are the accidental host who acquires infection by direct or indirect exposure to urine or other products of infected animals. It presents with a wide range of clinical manifestations from mild fever to severe hepatic and renal involvement. Due to a lack of clinical awareness, proper epidemiological data, and proper diagnostic facilities in resource-limited settings, leptospirosis still remains an underdiagnosed and underreported disease. This study aims to determine the seroprevalence of leptospirosis among suspected patients with acute febrile illness who attended the OPD or were admitted to wards of this tertiary care hospital in Northern Assam and to analyse the clinical pattern of disease occurrence.

Materials and methods

A hospital-based retrospective study was conducted in the Department of Microbiology, Tezpur Medical College and Hospital, Assam, from April 2023 to March 2024. A total of 1,372 serum samples, collected from acute febrile illness patients, were tested for *Leptospira* IgM antibody using a commercially available *Leptospira* IgM enzyme-linked immunosorbent assay (ELISA) kit, and the clinical pattern of disease occurrence among the positive cases was studied. Data were analyzed using descriptive statistics and the chi-square test.

Results

Out of 1,372 patients of acute febrile illness, 213 were tested positive for *Leptospira* IgM, with a seroprevalence of 15.5%. Most prevalent in 61-70 years of age, with no statistical difference between females (115/687; 16.7%) and males (98/685; 14.3%). The highest frequency occurred during October-December (64; 30.1%). The most common clinical presentation was fever with cough (43; 20.2%), followed by vomiting (31; 14.6%) and headache (30; 14.1%).

Conclusion

With a seroprevalence rate of 15.5%, leptospirosis has emerged as an important cause of acute febrile illness in this region. Awareness among the clinicians is crucial for early diagnosis, and in a resource-limited setup, serological assays like ELISA can be an effective diagnostic approach.

## Introduction

Leptospirosis is a zoonotic disease with worldwide distribution, more prevalent in tropical and subtropical countries. It has emerged as an important public health concern worldwide, including in India. It is caused by the spirochete *Leptospira* spp. [1]. It is estimated that the incidence of leptospirosis is over 10 lakh cases globally, and almost 60,000 deaths are reported annually [2]. In India, a total of 21,046 laboratory-confirmed cases of leptospirosis were reported, with 291 deaths and 57 outbreaks, with a case fatality rate of zero to 7% between 2015 and 2020. On average, 4,753 cases of leptospirosis are reported every year, with an average of 83 deaths [2]. The genus *Leptospira* is divided into two species, the pathogenic and the free-living *Leptospira*, viz. *Leptospira interrogans *and *Leptospira biflexa,* respectively. Serotypes of *Leptospira interrogans *are the agents causing human leptospirosis [3]. Rodents, particularly rats and mice, are the most common reservoir of infection along with other domestic animals, such as cattle, pigs, and dogs [4]. Humans are the accidental host who acquires the infection by direct or indirect exposure to urine or other products of infected animals. Risk factors include close proximity to infected animals, contact with infected waterbodies, poor sanitation, occupational risk among farmers, sewage workers, butchers, and leisure activities in contaminated water. However, reports of human-to human transmission are extremely rare [3,4]. It presents with a wide range of clinical manifestations, which include fever, headache, rash, myalgia, conjunctival suffusion, hepatosplenomegaly, haemorrhagic manifestations, and aseptic meningitis. It can also lead to hepatorenal involvement, termed as “icteric” infection, thereby resulting in jaundice. In severe cases, it leads to acute respiratory distress syndrome (ARDS) and pulmonary haemorrhage [3,5]. The patients with mild symptoms are usually treated with doxycycline, 100 milligrams, twice daily for seven days. In severe cases, intravenous penicillin G is the drug of choice, in the dose of 1.5 million units, six hourly, or ceftriaxone 1 gram twice daily for seven days. In case of allergy to these drugs, doxycycline, azithromycin, or clarithromycin may be used [6]. Preventive measures include chemoprophylaxis with doxycycline at a dose of 200 milligrams weekly, one week prior to and continued throughout the exposure period in high-risk individuals. Furthermore, rodent control, immunization through vaccination, and disinfection of contaminated water sources are other general measures [4,6].

Diagnosis of leptospirosis depends on clinical features and history of exposure. Laboratory diagnosis consists of isolation of *Leptospira* in culture, which is considered the gold standard, and by the polymerase chain reaction (PCR) technique. However, culture is time-consuming and needs a higher biosafety level, making it unsuitable for rapid diagnosis in the acute stage of illness. Although PCR has higher sensitivity and specificity, in a resource-limited setup, it is not a commonly used method. Serological tests are often the preferred method for the diagnosis of leptospirosis, which includes the MAT (microscopic agglutination test) and enzyme-linked immunosorbent assay (ELISA). Although MAT is considered the reference serological test, with higher specificity, it is technically demanding and time-consuming, and due to the need for live *Leptospira*, it is restricted to the reference laboratories [6]. Therefore, an alternative serological test for early diagnosis is the IgM ELISA. However, the lack of clinical awareness, proper epidemiological data, and lack of proper diagnostic facilities in a resource-limited set-up make it an underdiagnosed and underreported disease in the country. Therefore, the present study was undertaken to determine the seroprevalence of leptospirosis among suspected patients with acute febrile illness who attended the OPD or were admitted to the wards of Tezpur Medical College and Hospital, a tertiary care hospital in Northern Assam, and to analyze the clinical pattern of disease occurrence.

## Materials and methods

This hospital-based retrospective study was conducted in the Department of Microbiology, Tezpur Medical College and Hospital, Assam, between April 2023 and March 2024. The hospital caters to the population of Sonitpur district, as well as the nearby districts in the Northern bank of the Brahmaputra River. The population is largely dependent on the agriculture sector and the tea plantation. The study was approved by the Institutional Ethics Committee (Reg. no.: EC/NEW/INST/2022/1706; IEC Sl no.: 2024/064/TMC&H). A total of 1,372 serum samples, collected from acute febrile illness cases suspected of leptospirosis, as advised by clinicians for diagnosis and treatment, were included in the study.

Inclusion criteria

All patients suspected of leptospirosis who presented with acute febrile illness and attended the OPD or IPD of Tezpur Medical College and Hospital, Assam, were included in the study.

Exclusion criteria

The patients who were not willing to provide informed consent and those who tested positive for malaria, dengue, enteric fever, sepsis, and meningitis were excluded from the study.

Sample collection and processing

Three to five ml of blood samples were collected by designated laboratory technicians in a clot vial and were centrifuged at 3000 rpm for 10 minutes to separate the serum. Serum samples were aliquoted, properly labelled, and stored at -20 °C until tested. The samples were tested for *Leptospira* IgM antibody using a commercially available *Leptospira* IgM ELISA kit (*Leptospira* IgM Microlisa, J. Mitra & Co. Pvt. Ltd., New Delhi, India). The test was performed by designated laboratory technicians, according to the manufacturer’s instructions provided in the kit insert. With each run, along with the samples, positive and negative test controls were tested. The ELISA run was considered valid when the optical density (OD) of the negative control was <0.3, and that of the positive control was >1.1, as mentioned in the kit insert. For result interpretation, calculation of the cut-off value, sample O.D ratio, and *Leptospira* IgM units were done. The test results were interpreted as *Leptospira* IgM units, based on the manufacturer’s reporting protocol. *Leptospira* IgM units <9 were interpreted as negative for *Leptospira* IgM antibodies, equivocal between 9 and 11, and >11 as positive for *Leptospira* IgM antibodies.

For the *Leptospira* IgM antibody-positive cases, the demographic data, including age and gender, were collected from the test requisition form, and clinical history, presentation, and duration of illness were obtained from hospital records. The clinical pattern of disease occurrence among the positive cases of the *Leptospira* IgM was then analyzed.

Statistical analysis

Data were entered in Microsoft Excel sheets (Microsoft Corp., USA), and then the statistical analysis of the obtained data was done. The Chi-square test was done in Microsoft Excel to find out the p-values for determining the association between gender and disease status, as well as the association between age group and disease positivity. A p-value < 0.05 was considered significant. Data has been presented in appropriate tables and the p-values.

## Results

Of the 1,372 patients of acute febrile illness, 687 (50.1%) were female and 685 (49.9%) were male.

Out of the 1,372 patients, 213 tested positive for *Leptospira* IgM antibody, resulting in an overall seroprevalence of 15.5%. The prevalence among males and females was found to be 98/685 (14.3%) and 115/687 (16.7%), respectively. Statistically, no significant association between gender and disease status was found in this study, with p-value = 0.21 (>0.05) (Table 1).

**Table 1:** Gender-wise distribution of leptospirosis

Gender	Patients who tested positive	Patients who tested negative	Total population	p-value
Male	98 (14.3%)	587 (85.7%)	685 (100%)	0.21
Female	115 (16.7%)	572 (83.3%)	687 (100%)	
Total	213 (15.5%)	1,159 (84.5%)	1,372 (100%)	

The highest seropositivity of 17/67 (25.4%) was seen in the age group of 61-70 years, followed by 69/411 (16.8%) in 21-30 years, with a decline in positivity to 4/41 (9.8%) in individuals aged >71 years and lowest positivity in 2/38 (5.3%) children aged zero-10 years. No statistically significant association between age group and disease positivity was found in the study with p = 0.147(>0.05) (Table 2).

**Table 2:** Age-wise distribution of leptospirosis

Age group	Patients who tested positive	Patients who tested negative	Total population	p-value
0-10	2 (5.3%)	36 (94.7%)	38	0.147
11-20	34 (15.7%)	183 (84.7%)	217	
21-30	69 (16.8%)	342 (83.3%)	411	
31-40	34 (13.3%)	221 (86.7%)	255	
41-50	32 (16.4%)	163 (83.6%)	195	
51-60	21 (14.2%)	127 (85.8%)	148	
61-70	17 (25.4%)	50 (74.6%)	67	
>71	4 (9.8%)	37 (90.2%)	41	
Total	213	1,159	1,372	

The highest proportion of cases was observed during October-December (64; 30.1%), followed by July-September (60; 28.2%), indicating a possible seasonal trend (Table 3).

**Table 3:** Seasonal distribution of leptospirosis-positive cases

Months	Positive cases (%)
January- March	41 (19.2%)
April-June	48 (22.5%
July-September	60 (28.2%)
October-December	64 (30.1%)
Total	213

Among all the fever cases that tested positive for leptospirosis, the most common clinical presentation was cough (43; 20.2%), followed by vomiting (31; 14.6%) and headache (30; 14.1%) (Table 4). No mortality was reported among the patients during the study period.

**Table 4:** Clinical presentation of leptospirosis-positive cases

Clinical Presentation	Number of positive cases	Percentage (%)
Fever	213	100%
Cough	43	20.2%
Vomiting	31	14.6%
Headache	30	14.1%
Nausea	27	12.7%
Jaundice	22	10.3%
Myalgia	19	8.9%
Breathlessness	17	8%
Abdominal pain	16	7.5%
Sore throat	14	6.6%
Diarrhoea	10	4.7%
Arthralgia	3	1.4%
Irritability	1	0.5%

## Discussion

In the present study, the seroprevalence of leptospirosis was found to be 15.5%, which is comparable to the 13.13% reported from Kolkata, by Kumari P et al. in 2023 [7]. Closely aligned findings were observed in studies by Deshmukh P et al. [8], Baveja B. et al. [9], and Kumar PBP et al. [10], with seroprevalence rates of 12.7%, 19.78%, and 17%, respectively. However, higher prevalence rates of 39% and 24.5% were reported in the studies by Sen S S et al. [5] in South East Assam and by Kumar A et al. [1] in Jharkhand. By contrast, a lower prevalence rate of 2.13% was reported in a study by Malakar M et al. [11] from Lakhimpur and Dhemaji Districts of Assam. The observed differences in the seroprevalence rates of leptospirosis in different studies may be attributed to the different climatic conditions of different geographical locations. In addition, ecological factors such as rainfall, flooding patterns, farming practices, and rodent density vary across regions, which may significantly influence disease transmission, thus contributing to the variations in the prevalence rates in different studies.

The prevalence among females was found to be slightly higher than that among males, which was 115/687 (16.74%) and 98/685 (14.30%), respectively. However, statistically, no significant association between gender and disease status was found in this study, with a p-value = 0.21 (>0.05). Several studies have reported a significantly higher prevalence of the disease among males, likely due to increased occupational and environmental exposure, while a few have documented female predominance. A study conducted by Chitkara S et al. [12] reported a higher prevalence in males (80%). Similarly, male predominance of 65.44% and 57.00% was found in studies conducted by Chauhan R et al. [13] and Mandal MD et al. [14], respectively, indicating that males were more prone compared to females. Contrary to these, a study conducted by Deshmukh P et al. [8] reported seropositivity more in females (14%) than males (11.5%). Another study from Central India by Baveja B et al. [9] reported higher positivity among females (66%). In an international study from Nepal by Regmi L et al. [15], the male-to-female ratio was found to be 1:1.1 (25.0% females, 17.1% males), but no significant difference was found in the presence of leptospirosis among both sexes. The observed female predominance in these studies aligns with the findings of our study, although this difference did not reach statistical significance in the present study. This suggests that gender may not be an independent determinant of disease occurrence in our study population. This observation, while not sufficient to establish a true gender-based difference, suggests that the observed variation could be attributed to comparable exposure risks among males and females, and healthcare-seeking behaviour among both sexes. There is also a change in socio-occupational roles where traditional male predominance is diminishing due to males engaged in non-agricultural occupations such as construction work, transportation, whereas increased female participation in agricultural activities, livestock care, and outdoor household chores, washing clothes in contaminated water, cleaning flooded homes particularly during the monsoon and post-monsoon periods, that may equalizes disease risk thereby narrowing the traditional gender gap.

The study population ranged from zero to over 71 years of age. The disease positivity varied across age groups, with the highest seropositivity of 17/67 (25.4%) in the age group of 61-70 years, followed by 69/411 (16.8%) seen in 21-30 years, and the lowest of 2/38 (5.3%) in children aged zero-10 years. A decline in positivity rate to 4/41 (9.8%) was seen among the age group of >71 years. No statistically significant association between age group and disease positivity was found in the study. A study by Kumar A. et al. [1] reported the highest seroprevalence of 34.69% in the age group of 21‑40 and 41‑60 years. Similarly, the highest seropositivity of 29.3% in 21-40 years of age, followed by 18.6% in 51-60 years of age, was reported by Kumari P et al. [7]. Although there is no significant association of age group and disease positivity in this study, the slight differences reported among the various age groups may require further investigation.

Seasonal variation in seroprevalence was observed and found to be highest during October-December (64; 30.1%), followed by July-September (60; 28.2%). A study conducted by Kumar A et al. [1] reported the highest seroprevalence in October (57.14%). This region of the country has a sub-tropical humid climate, receiving heavy rainfall between June and September. The months of October and November are a period that follows heavy rainfall, causing the rivers to overflow. This may be attributed to increased exposure during the post-monsoon period, when water stagnation and environmental contamination persist despite declining rainfall. This period is also the main harvest season in this region of the country, when the risk of disease exposure increases.

The present study revealed a broad spectrum of symptoms, reflecting the multisystem nature of the disease. The commonest clinical presentation is fever with cough (43; 20.2%), followed by vomiting (31; 14.6%) and headache (30; 14.1%). In a substantial proportion of patients, the gastrointestinal symptoms, including nausea, vomiting, abdominal pain, and diarrhoea, were observed. In 22 (10.3%) cases, jaundice was noted, indicating hepatic involvement. Less common manifestations, such as arthralgia and irritability, highlight the variability in clinical presentation. These symptoms are almost consistent with previous studies. A study by Srijith K et al. [16] in Thrissur, Kerala, reported myalgia, jaundice, cough, and breathlessness among the most common presentations. Another study by Kumari P et al. [7] in 2023 reported headache, followed by myalgia and jaundice.

The strength of the present study lies in providing an insight into the seroprevalence and clinical profile of leptospirosis-positive patients, thus helping to understand the pattern of disease occurrence. The study also emphasizes the use of ELISA for the detection of *Leptospira* IgM antibody as an effective diagnostic approach. However, the present study has certain limitations. Being a hospital-based study, the findings could not generalise the true prevalence of leptospirosis in the community, as only patients seeking medical care were part of the study population. In addition, serological diagnosis was done by a genus-specific test, IgM ELISA for *Leptospira*. Due to resource-limited factors, the serovar-specific MAT (microscopic agglutination test), which is the standard reference test, could not be performed. As this was a retrospective study based on hospital records, information on occupation, socioeconomic status, and laboratory parameters could not be included in the study.

## Conclusions

Leptospirosis has emerged as an important cause of acute febrile illness in this region, with an overall seroprevalence rate of 15.5%, as observed in the present hospital-based study. A high positivity rate was observed in the post-monsoon months, indicating a seasonal trend. However, no statistically significant association was found between gender and disease occurrence. Although a certain age group showed higher positivity, it was not found to be statistically significant. The clinical presentations of Leptospirosis were often varied and nonspecific, which can resemble other acute febrile illnesses. Awareness among clinicians is crucial for early suspicion and timely laboratory testing for an early diagnosis and treatment initiation. This, in turn, will reduce the morbidity and late complications of the disease. In a resource-limited setup, serological assays like ELISA offer a feasible and effective diagnostic approach. A larger community- based study is needed for better evaluation of the disease burden, its associated risk factors, and formulation of effective preventive strategies at the community level.
